# Artificial intelligence and surgery

**DOI:** 10.1002/ags3.12766

**Published:** 2023-12-18

**Authors:** Masashi Takeuchi, Yuko Kitagawa

**Affiliations:** ^1^ Department of Surgery Keio University School of Medicine Tokyo Japan

Artificial intelligence (AI) has a significant impact on the field of health care, particularly imaging and video analyses. It can considerably support clinical decision‐making, including the automatic diagnosis of gastrointestinal cancer during endoscopy and automated detection of pulmonary lesions on computed tomography (CT).^1^, ^2^ In the future, AI may provide innovative solutions that improve surgical efficiency and patient outcomes in the field of surgical procedures. Integrating AI into surgery can potentially redefine the surgical procedures, ushering in a new era of personalized and data‐driven healthcare.

## 1 AI IN PREOPERATIVE PLANNING

The role of AI in preoperative planning is substantial. By analyzing large amounts of medical data, including patient records, imaging findings (e.g., CT and endoscopy), and previous history, AI can help surgeons plan more effective and personalized surgical strategies. For instance, it can predict potential perioperative complications, suggesting optimal surgical approaches, and even simulate surgical outcomes. Furthermore, AI can analyze CT scans to create 3D models of patient anatomy, such as blood vessels, allowing surgeons to plan surgeries with a level of detail that was previously unattainable.

## 2 ROBOTIC SURGERY AND AI NAVIGATION

Robotic surgery, one of the most notable applications of AI in surgery, has been a game changer. Compared with the conventional approach, robotic systems such as the da Vinci Surgical System enable surgeons to perform fine and complex procedures with more precision, flexibility, and control. AI enhances these systems by providing real‐time analysis and precision in movement and learning from each surgery performed, thereby improving the outcomes over time. In particular, advanced image recognition powered by AI algorithms aids in quickly identifying critical anatomical structures, navigating complicated anatomy more easily, and reducing the risk of developing surgical complications.^3^, ^4^ In addition, AI's ability to understand and analyze surgical process facilitates seamless information sharing across the surgical team, contributing to the early detection of complications and ensuring rapid intervention.^5^ Moreover, incorporating AI into surgical robots represents a giant leap forward. These AI algorithm‐controlled robotic systems perform complex procedures with unparalleled precision. They are often tailored to adapt to the unique skills of individual surgeons, thereby improving surgical accuracy while minimizing the potential for error. The synergistic cooperation between human surgeons and AI‐guided robotic systems represents a new era of cooperative, high‐leveled surgery.

## 3 POSTOPERATIVE CARE AND AI

AI also plays a pivotal role in overseeing and optimizing patient recovery after surgery. Through the use of sensors and data analysis, AI can foresee potential complications and readmissions before serious problems develop, enabling surgeons to provide timely and targeted interventions and contributing significantly to overall postoperative care. AI involvement goes beyond mere monitoring to tailoring postoperative care plans through a high degree of personalization based on each patient's unique data profile. This personalized approach considers individual health parameters, medical history, and specific recovery needs. Such precision in care planning addresses patient‐specific requirements and improves the overall recovery experience, thereby potentially accelerating the healing process.

## 4 AI IN SURGICAL EDUCATION

AI is also revolutionizing surgical training for trainees. Implementing AI in training includes the use of innovative tools such as virtual reality and augmented reality simulations, fundamentally changing the learning experience of surgeons. With AI able to tailor the training experience to each trainee's skill level, these simulations can provide a dynamic and adaptive learning platform that goes beyond traditional methods; thus, the modules can be individualized according to the trainee's needs and proficiency level.

In this way, young surgeons can immerse themselves in real scenarios and gain hands‐on experience without the risks associated with actual surgery. By utilizing AI‐enhanced interactive simulations, surgical techniques can be thoroughly understood, laying the foundation of practical skills essential for successful surgery.

## 5 ETHICAL AND PRACTICAL CONSIDERATIONS

While AI demonstrates significant benefits in surgery, it still has ethical and practical challenges. Reliance on AI and robotic systems raises questions about liability and accountability in the event of surgical errors. Given that these systems require access to patients' personal data, data privacy and security issues also emerge. Training surgeons to effectively use these advanced technologies is also a challenge because of the need for equitable access to these technologies among different geographic and socioeconomic groups.

In conclusion, the integration of AI into surgery represents a major leap forward in medical technology. It can potentially increase surgical accuracy, improve patient outcomes, and revolutionize surgical training. However, the ethical and practical challenges associated with AI must be addressed. With careful consideration and continued development, AI in surgery will continue to evolve and benefit patients and healthcare professionals.

## CONFLICT OF INTEREST STATEMENT

Yuko Kitagawa received lecture fees from Chugai Pharmaceutical Co., Ltd., Taiho Pharmaceutical Co., Ltd, Asahi Kasei Pharma Corporation, Otsuka Pharmaceutical Factory Inc., Shionogi & Co., Ltd., Nippon Covidien Inc., Ono Pharmaceutical Co., Ltd., Bristol‐Myers Squibb K. K. Author Y. K was supported by grants from Chugai Pharmaceutical Co., Ltd., Taiho Pharmaceutical Co., Ltd, Yakult Honsha Co. Ltd., Asahikasei Co., Ltd., Otsuka Pharmaceutical Co., Ltd., Takeda Pharmaceutical Co., Ltd., Ono Pharmaceutical Co., Ltd., Tsumura & Co., Kyouwa Hakkou Kirin Co., Ltd., Dainippon Sumitomo Pharma Co., Ltd., EA Pharma Co., Ltd., Astellas Pharma Inc., Toyama Chemical Co., Ltd., Medicon Inc., Kaken Pharmaceutical Co., Ltd., Eisai Co., Ltd., Otsuka Pharmaceutical Factory Inc., Teijin Pharma Ltd., Nihon Pharmaceutical Co., Ltd., and Nippon Covidien Inc. Author Y. K held an endowed chair provided by Chugai Pharmaceutical Co., Ltd., and Taiho Pharmaceutical Co., Ltd, outside the submitted work. Masashi Takeuchi has stocks from Direava inc. outside the submitted work. Author Yuko Kitagawa is a current chief editor of *Annals of Gastroenterological Surgery*.
